# Traumatic Brain Injury Causes Aberrant Migration of Adult-Born Neurons in the Hippocampus

**DOI:** 10.1038/srep21793

**Published:** 2016-02-22

**Authors:** Sara Ibrahim, Weipeng Hu, Xiaoting Wang, Xiang Gao, Chunyan He, Jinhui Chen

**Affiliations:** 1 Spinal Cord and Brain Injury Research Group, Department of Neurosurgery, Stark Neuroscience Research Institute, Indianapolis, Indiana, United States of America; 2 Department of Neurosurgery, 2nd Affiliated Hospital, Fujian Medical University, Quanzhou, 362000, China; 3 School of Biomedical Sciences, Huaqiao University, Quanzhou, 362000, China

## Abstract

Traumatic brain injury (TBI) promotes neural stem/progenitor cell (NSC) proliferation in an attempt to initiate innate repair mechanisms. However, all immature neurons in the CNS are required to migrate from their birthplace to their final destination to develop into functional neurons. Here we assessed the destination of adult-born neurons following TBI. We found that a large percentage of immature neurons migrated past their normal stopping site at the inner granular cell layer (GCL), and became misplaced in the outer GCL of the hippocampal dentate gyrus. The aberrant migration of adult-born neurons in the hippocampus occurred 48 hours after TBI, and lasted for 8 weeks, resulting in a great number of newly generated neurons misplaced in the outer GCL in the hippocampus. Those misplaced neurons were able to become mature and differentiate into granular neurons, but located ectopically in the outer GCL with reduced dendritic complexity after TBI. The adult-born neurons at the misplaced position may make wrong connections with inappropriate nearby targets in the pre-existing neural network. These results suggest that although stimulation of endogenous NSCs following TBI might offer new avenues for cell-based therapy, additional intervention is required to further enhance successful neurogenesis for repairing the damaged brain.

Traumatic brain injury (TBI) is the leading cause of death in children and young adults^1^. TBI primarily causes cell death in the cortex. In addition, it induces secondary cell death in the hippocampus, leading to substantial cognitive impairment, motor dysfunction, and often epilepsy^2^. In the past two decades, our understanding of the cellular and molecular changes that occur following TBI have significantly increased. Although some of the injury is due to the primary mechanical events (i.e. tearing of nerve cells and blood vessels)^3^,^4^,^5^, the majority of post-traumatic cell death is due to a cascade of secondary events occurring in the first minutes, hours and days following TBI. These secondary events compound the primary injury^4^,^6^,^7^, leading to further cognitive, sensory, and motor dysfunction^4^,^5^,^6^,^7^,^8^. Hippocampal-associated learning and memory impairment is one of the most significant residual deficits following TBI, and is one of the most frequent complaints heard from patients and their relatives^9^,^10^,^11^. Due in part to the lack of understanding of the cellular and molecular mechanisms that lead to secondary cell death, effective treatment options are nonexistent.

The discovery of neural stem/progenitor cells (NSCs) in the adult brain offers a potentially promising strategy for repairing CNS injury following TBI. Other groups and our previous study showed that TBI promotes NSC proliferation in an attempt to initiate innate repair and/or plasticity mechanisms^12^,^13^,^14^,^15^,^16^,^17^,^18^,^19^. However, the spontaneous posttraumatic recovery of hippocampal-related cognitive and memory function is limited. These results suggest that although stimulation of endogenous adult NSCs following TBI might offer new avenues for cell-based therapy, a better understanding of neurogenesis (birth of neurons) following TBI is required to further enhance successful neurogenesis for repairing the damaged brain.

In the adult hippocampus, NSCs reside in the subgranular zone (SGZ), and give rise to newborn neurons there. These newborn neurons initially locate in the SGZ, migrate radially from there into the granular cell layer (GCL), mostly reside in the inner one third of the GCL, and integrate functionally into the existing neural network^20^,^21^,^22^,^23^,^24^. The neurogenesis is a long process at least including NSC proliferation, differentiation, migration, polarization, axonal and dendritic specification, axonal guidance, maturation, and synaptogenesis of newborn neurons. Our previous studies found that TBI affected multiple steps in the process of neurogenesis, including promoting NSC proliferation^25^, inducing newborn neuron death^26^, and impairing dendrite development of neurons in the GCL^27^,^28^,^29^. It is still not known whether TBI affects neuronal migration, which is one of the fundamental processes for new neurons to reach their proper positions and integrate into the neural network with appropriate targets^30^. Thus, we assessed whether the newborn neurons migrate to their proper positions following TBI.

## Results

### TBI caused aberrant migration of newborn immature neurons in the hippocampus

**Figure 1:**
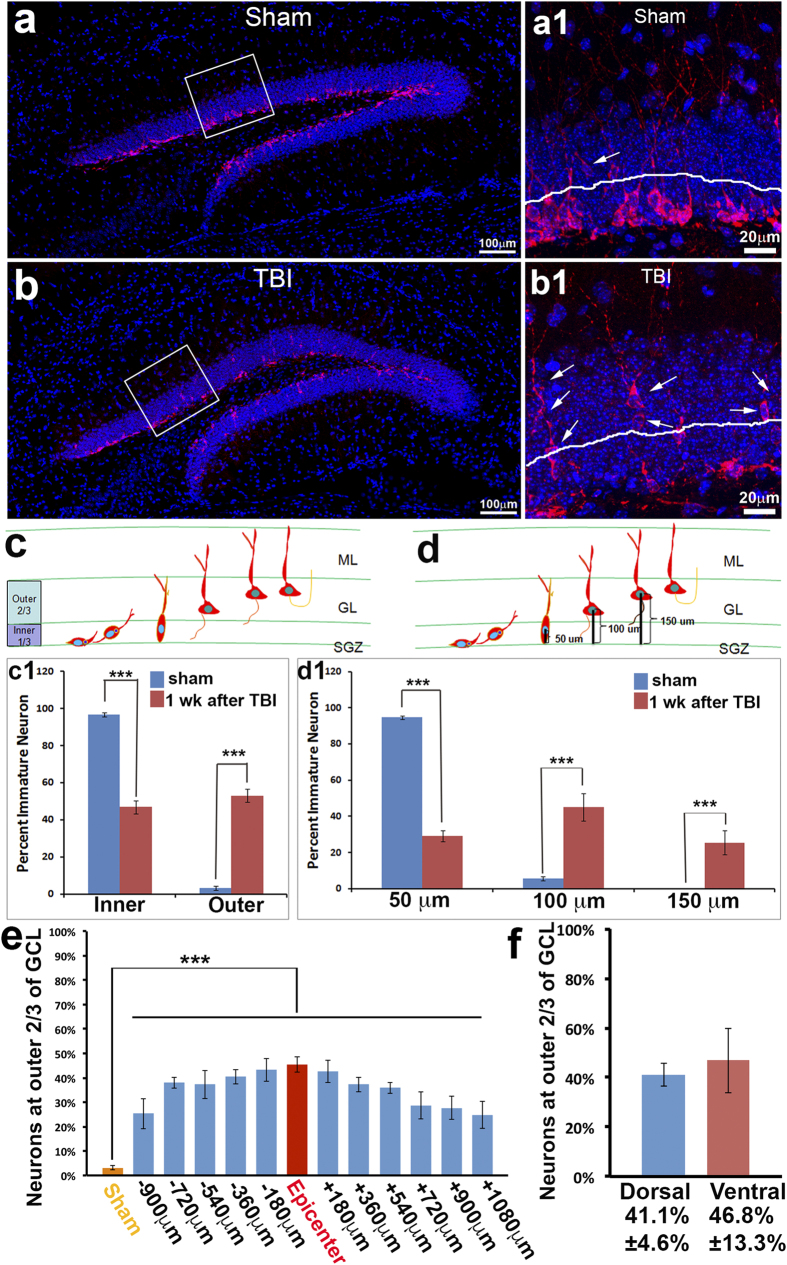
TBI caused aberrant migration of newborn immature neurons in the hippocampus. Mice at an age of 12 weeks received either moderate controlled cortical impact (CCI) (n = 6) or sham surgery (n = 6). One week after injury, the brains were removed for immunostaining with antibody against doublecortin (Dcx). (**a**) Positions of adult-born immature neurons labeled by specific marker Dcx (red) in the hippocampus of sham-treated mice. (a1) High power image shows that most of Dcx-positive cells line up in the inner layer of the granular cell layer (GCL). (**b**) Positions of adult-born immature neurons in the hippocampus of TBI-injured mice at 1 week following surgery. (b1) High power image shows that many Dcx-positive cells aberrantly migrated out to the outer layer of the GCL (arrows). (**c**) Cartoon to illustrate the method used for quantifying the position of immature neurons in the hippocampal dentate gyrus. Neurons are measured as either residing in the inner one third or the outer two thirds of the GCL. (c1) Comparison of sham and TBI percentage of immature neurons residing in either the inner one third or the outer two thirds of the granular cell layers at the 1-week time point. (**d**) Cartoon to illustrate the method used for quantifying the migration distance of immature neurons in the hippocampal dentate gyrus. Migration is measured in three categories: within 50 μm, 50–100 μm, and beyond 100 μm. (d1). Comparison of sham and TBI percentage of immature neurons migrating within the three categories of migration (***p < 0.001). (**e**). Quantification of the distribution of displaced immature neurons in the whole hippocampal dentate gyrus 1 week after TBI. (**f**). Comparing the displaced immature neurons between the dorsal and ventral blades of hippocampal dentate gyrus 1 week after TBI.

In the adult hippocampus, new neurons were born in the SGZ and migrated radially with a very short distance into the GCL, stop migration, and, finally, most of them located in the inner one-third of the GCL^20^,^21^,^22^,^23^,^24^. To determine whether the newborn neurons migrate to their proper position in the hippocampal dentate gyrus (HDG) following TBI, we assessed the positions of newborn neurons one week after TBI. Mice received either moderate controlled cortical impact (CCI) injury (n = 6) or sham surgery (n = 6). One week after injury, the mice were sacrificed. The brains were removed for immunostaining with antibody against doublecortin (Dcx), a marker widely used for detecting immature neurons^31^,^32^. Dcx-positive immature neurons (Fig. 1, red) in the hippocampus of sham-surgery mice mainly located at the inner granular cell layer (GCL) of the hippocampal dentate gyrus (HDG) (Fig. 1a) with their dendrites projecting toward the molecular layer (ML) (Fig. 1a1). When we divided the GCL into the inner one-third and outer two-thirds (white line shown in Fig. 1a1,b1, and cartoon in Fig. 1c), most of the immature neurons were located in the inner one-third, a few cells were located in the outer two-thirds of the GCL (indicated by white arrow in Fig. 1a1), and only occasionally Dcx-positive cells were observed in the hilus. Further quantification of the immature neurons in the GCL showed that 96.8 ± 0.4% of immature neurons resided in the inner one-third, and only 3.2 ± 0.4% immature neurons located in the outer two-thirds of the GCL in the sham-surgery mice (Fig. 1c1). These data indicated that newborn neurons were guided to migrate from their birthplace at the SGZ into the GCL. Most of the immature neurons migrated a short distance towards the GCL and stopped at the inner one-third of the GCL; only a small portion of them migrated further entering the outer two-thirds zone of the GCL. This recapitulates many reports about neuronal migration in the adult hippocampus^33^,^34^. When we assessed the immature neurons in the hippocampus of TBI-injured mice, we found that the number of Dcx-positive immature neurons reduced from 40.14 ± 2.73/mm to 17.4 ± 2.3/mm, compared to sham control (Fig. 1b). There are obviously many more immature neurons located in the outer two-thirds of the GCL (Fig. 1b1, indicated by white arrows). Quantification showed that 46.9 ± 3.5% of immature neurons resided in the inner one-third of the GCL. In contrast, the percentage of immature neurons located in the outer two-thirds of the GCL increased to 53.1 ± 3.5%, indicating that a significant number of immature neurons did not stop migrating before reaching the inner one-third of the GCL, and they migrated ectopically into the outer two-thirds of the GCL in the hippocampus 1 week following TBI.

We assessed the migration distance of each immature neuron by measuring the distances between the center of their cell bodies and the borderline of the subgranular zone and the hilus in sham mice (Fig. 1d1). We observed that 94.6 ± 1.0% of immature neurons migrated less than 50 μm, and only 5.4 ± 1.0% of immature neurons migrated more than 50 μm, but none of them migrated more than 100 μm. In contrast, 1 week after TBI, only 29.4 ± 3.0% of immature neurons had migrated less than 50 μm, 45.1 ± 7.5% of them migrated 50–100 μm, and 25.6 ± 6.5% migrated even more than 100 μm.

We further assessed the distribution of the displaced immature neurons across the whole hippocampus 1 week after TBI (Fig. 1e). We found that the epicenter has the highest number of displaced immature neurons in the outer two-thirds of the GCL. The number of displaced immature neurons in the outer two-thirds of the GCL decreased slightly in both rostral and caudal directions, and was observable almost across the whole hippocampus. The dentate gyrus appears as a V-shaped structure, composed of a dorsal blade and a ventral blade. There is no difference in the number of displaced immature neurons in the outer two-thirds of the GCL between the dorsal and ventral blades of the dentate gyrus (Fig. 1f).

Together, these data indicated that TBI causes the aberrant migration of immature neurons and the misplacement of a significant number of them in the outer GCL of the hippocampus.

### The aberrant migration of immature neurons lasts for weeks following TBI

**Figure 2:**
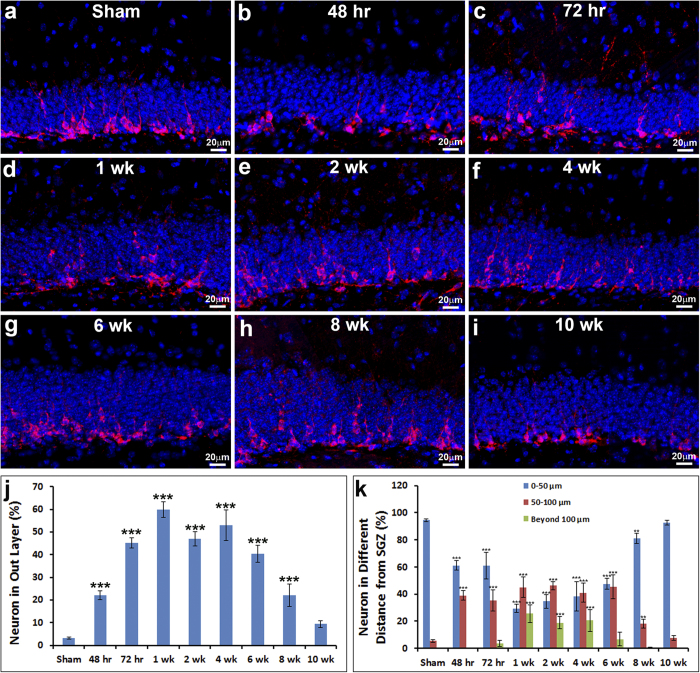
The aberrant migration of immature neurons after TBI lasts about 8 weeks. Mice at an age of 12 weeks received either moderate controlled cortical impact (CCI) or sham surgery (n = 5 each group). The brains were removed for immunostaining with antibody against doublecortin (Dcx) to exhibit the positions of immature neurons right after sham-surgery (**a**), 48 hr, (**b**), 72 hr (**c**), 1 wk (**d**), 2 wk (**e**), 4 wk (**f**), 6 wk (**g**), 8 wk (**h**), or 10 wk (**i**) after TBI respectively. (**j**) Percentage of immature neurons that located at the outer two-thirds of granular cell layer at different time after surgery. (**k**) Migratory distances of immature neurons in the hippocampal dentate gyrus at different time points after surgery.

We further investigated when the aberrant migration occurred and how long it lasted by assessing the position of immature neurons in the HDG at different times after TBI. The mice received moderate CCI (n = 40) and were sacrificed at 48 hours, 72 hours, 1 week, 2 weeks, 4 weeks, 6 weeks, 8 weeks, and 10 weeks following TBI (n = 5 for each group). The immunostaining with Dcx was performed to detect the positions of immature neurons (Fig. 2). We used the same quantitative approach to assess the immature neurons positioned at either the inner one-third or outer two-thirds of the GCL of HDG.

At 48 hours after TBI, 22.1 ± 2.0% of immature neurons were located in the outer two-thirds of the GCL. The increase of immature neurons in the outer two-thirds of the GCL was significantly different from the sham control (p < 0.001). At 72 hours following TBI, the percentage of immature neurons that had located in the outer two-thirds of the GCL further increased to 45.3 ± 2.2% and peaked at 53.1 ± 3.5% at 1 week. The percentage of immature neurons located in the outer two-thirds of the GCL graduated reduced thereafter. At 2 weeks after TBI, 48.4 ± 3.1% of immature neurons were located in the outer two-thirds of the GCL; at 4 weeks, 53.0 ± 6.7%; at 6 weeks, 40.5 ± 3.7%; and at 8 weeks, 22.1 ± 4.9%; these percentages were all significantly greater than the control mice. At 10 weeks after TBI, the percentage of immature neurons located in the outer two-thirds of the GCL reduced to 9.4 ± 1.5%. The difference was not statistically significant in comparison to the control mice.

We further measured the migration distance of individual neurons in mice receiving sham-surgery or TBI. In the sham-surgery mice we found that 94.5 ± 4.9% of immature neurons migrated less than 50 μm, and only 5.5 ± 0.1% migrated 50–100 μm, while none migrated more than 100 μm. In contrast, at 48 hours after TBI, only 61 ± 3.5% of immature neurons migrated less than 50 μm; 39 ± 3.6% migrated 50–100 μm; and none migrated more than 100 μm. At 72 hours after TBI, 60.9 ± 3.5% of immature neurons migrated less than 50 μm; 35.4 ± 7.6% migrated 50–100 μm; and 3.8 ± 2.2% migrated more than 100 μm. At 1 week after TBI, 29.3 ± 1.7% of immature neurons migrated less than 50 μm; 45.1 ± 7.5% migrated 50–100 μm; and 25.6 ± 6.5% migrated more than 100 μm. At 2 weeks after TBI, 34.7 ± 4.0% of immature neurons migrated less than 50 μm; 46.3 ± 3.1% migrated 50–100 μm; and 19.0 ± 4.6% migrated more than 100 μm. At 4 weeks after TBI, 38.4 ± 5.1% of immature neurons migrated less than 50 μm; 41.1 ± 6.9% migrated 50–100 μm; and 20.5 ± 8.3% migrated more than 100 μm. At 6 weeks after TBI, 45.7 ± 6.7% of immature neurons migrated less than 50 μm; 45.5 ± 8.8% migrated 50–100 μm; and 6.8 ± 4.9% migrated more than 100 μm. At 8 weeks after TBI, 81.2 ± 11.1% of immature neurons migrated less than 50 μm; 18.3 ± 3.4% migrated 50–100 μm; and 0.5 ± 0.5% migrated more than 100 μm. At 10 weeks after TBI, 92.5 ± 9.8% of immature neurons migrated less than 50 μm; 7.5 ± 1.8% migrated 50–100 μm; and none migrated more than 100 μm. The migration distance of immature neurons at 10 weeks after TBI was comparable to the migration distance of immature neurons in the sham-surgery mice. The difference was no longer statistically significant.

These data indicated that the aberrant migration occurred as early as 48 hours after TBI and it lasted at least 8 weeks. It is worth noticing that about half of the immature neurons aberrantly migrated to the outer two-thirds of the GCL from 72 hours to 6 weeks after TBI. Altogether, the data showed that TBI significantly caused misplacement of immature neurons in the hippocampus for an extended period of time.

### Post-injury born misplaced neurons could mature at the ectopic position

**Figure 3:**
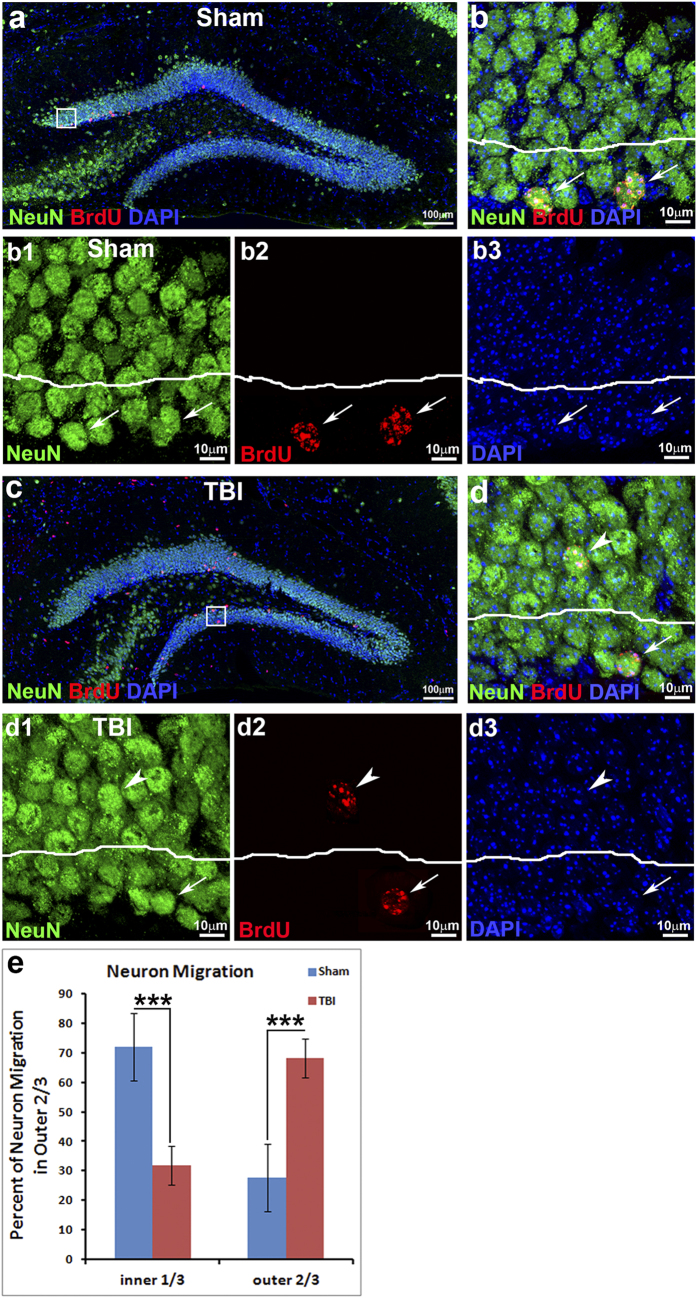
TBI results in misplacement of mature neurons born after injury. Mice at an age of 12 weeks received either moderate controlled cortical impact (CCI) or sham surgery (n = 5 each group). The mice then received an injection of 5-bromo-2′-deoxyuridine (BrdU, i.p. 50 mg/kg) once a day for 7 consecutive days to label the cells born within this time period. The mice were sacrificed 4 weeks after the last BrdU injection for determining the position of newborn mature neurons in the hippocampal dentate gyrus by double immunostaining with antibodies against BrdU and NeuN (Neuronal Nuclei). (**a**) Positions of adult-born neurons co-labeled by BrdU (red) and mature neuron marker NeuN (green) in the hippocampus of sham-treated mice. (**b**) High power magnification of image a (white box) shows that double-labeled neurons sit in the inner layer of the granular cell layer (GCL, white arrows). (b1-b3) Images of single focal section to show co-labeling of NeuN (green) with BrdU (red) in inner GCL in panel b (white arrows). (**c**) Positions of adult-born neurons in the hippocampus of TBI-injured mice at 5 weeks following surgery. (**d**) High power magnification of image c (white box) shows some of the mis-located BrdU and NeuN double-labeled neurons in the outer layer of the GCL (white arrowheads) in TBI mice. (d1-d3) Images of single focal section to show co-labeling of NeuN (green) with BrdU (red) in inner GCL (white arrows) and outer GCL (white arrowheads) in panel d. DAPI (blue) staining shows structure of hippocampal dentate gyrus. (**e**) Quantification of newborn mature neurons in the inner one-third or outer two-thirds of the GCL 4 weeks after last BrdU injection. (***p < 0.001).

In order to determine whether aberrant migration of immature neurons further resulted in misplacement of the mature neurons in the hippocampus following TBI, we labeled the cells born at the first weeks following TBI or sham-surgery as control with 5-bromo-2′-deoxyuridine (BrdU). Since it takes about 4 weeks for adult-born neurons to develop mature neuronal morphology and express mature neuronal markers^35^,^36^,^37^, we waited 4 weeks after the last BrdU injection to let the immature neurons mature before sacrificing the mice. Double immunostaining with antibodies against BrdU and NeuN (Neuronal Nuclei), a widely used marker for mature neurons, was performed to assess the positions of mature neurons born during the first week after TBI. The results showed that the surviving BrdU-positive cells expressed NeuN (Fig. 3a,b,b1–b3), indicating that in the sham-surgery mice the new neurons, born during the first week after TBI, had matured. The BrdU and NeuN double positive cells mostly (97.48 ± 1.54%) located at the inner one-third of the GCL, and only 2.52 ± 1.53% of them located in the outer two-thirds of the GCL of the sham-surgery mice (Fig. 3e). In contrast, only 46.96 ± 6.88% of BrdU and NeuN double positive cells located at the proper position at the inner one-third of the GCL in the TBI-injured mice (Fig. 3c,d,d1–d3). The BrdU and NeuN double positive cells in the outer two-thirds of the GCL dramatically increased to 53.04 ± 6.89%; this difference was statistically significant (P < 0.001). These data demonstrated that TBI significantly induced the misplacement of post-injury-born mature neurons at the outer GCL.

### The misplaced neurons born after injury can still specify to the right type of neurons in the hippocampus

**Figure 4:**
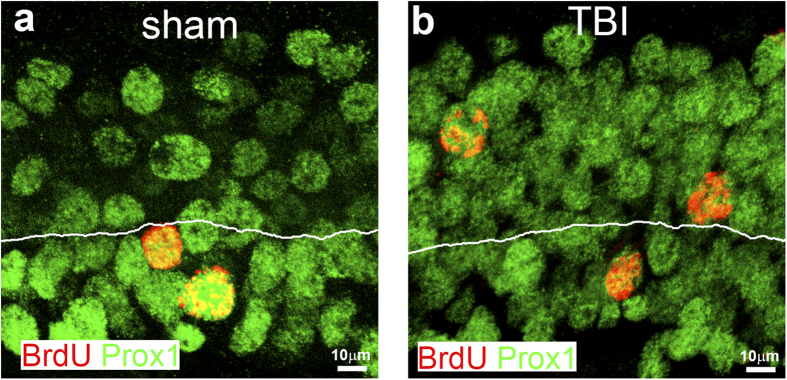
The misplaced neurons born after injury can still specify to right type of neurons in the hippocampus. Mice at an age of 12 weeks received either sham surgery (**a**) or moderate controlled cortical impact (CCI) (**b**) (n = 5 each group). The mice then received an injection of 5-bromo-2′-deoxyuridine (BrdU, i.p. 50 mg/kg) once a day for 7 consecutive days to label the cells born within this time period. The mice were sacrificed 4 weeks after the last BrdU injection for determining the neuron subtype specification of mature neurons in the hippocampal dentate gyrus produced at the first week after TBI with double immunostaining with antibodies against BrdU and Prox1. (**a**) The mature neurons in the hippocampal dentate gyrus produced in the first week after sham-surgery located at the inner one-third of the granular cell layer (GCL) labeled by both BrdU and Prox1. (**b**) The mature neurons in the hippocampal dentate gyrus produced at the first week after TBI located at the inner one-third and the outer two-thirds of the GCL labeled by both BrdU and Prox1.

Numerous subtypes of neurons exist in the adult brain. New neurons differentiate into specific types of neurons after they are born. Adult-born neurons in the hippocampus are normally specified to granular neurons with Prox1 expression^38^. To assess whether the misplaced neurons in the outer GCL still differentiate into granular neurons, we examined the mature neurons born during the first week after TBI. Mice received either TBI injury or sham-surgery, and then received an injection of BrdU once a day for 7 consecutive days, to label the new cells born during this period. Further more, double immunostaining with antibodies against BrdU and Prox1 was performed to assess whether the newborn neurons still specify into hippocampal granular neurons. The results showed that the BrdU positive cells mainly located at the inner GCL and expressed Prox1 in the hippocampus of sham-surgery control mice (Fig. 4a). In contrast, the BrdU positive cells in the GCL of TBI mice located both in the inner layer and the outer layer. Despite their different locations in the GCL, both of them expressed Prox1 (Fig. 4b). These data indicated that the misplaced neurons born after injury can still specify to the right type of neurons in the hippocampus.

### Neuron misplacement impairs their morphological development

**Figure 5:**
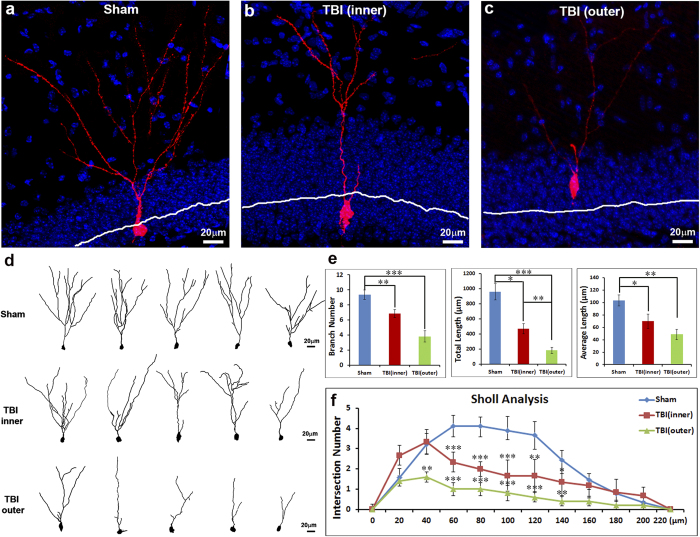
Neuron misplacement impairs their morphological development. Mice at an age of 12 weeks received an injection of retroviruses expressing red fluorescent protein (RFP) transcranially into the hippocampal hilus to label the dividing cells and trace their fate based on the RFP expression. One day after viral injection, the mice received either sham surgery or moderate controlled cortical impact (CCI). Five weeks after TBI, the mice were sacrificed to assess the dendrite arborization of RFP expressing neurons. (**a**) RFP labeled adult-born neurons in the hippocampus of sham-treated mice. (**b**) RFP labeled adult-born neurons in the inner layer of the hippocampus of TBI mice 5 weeks after injury. (**c**) RFP labeled mis-located adult-born neurons in the outer two-thirds layer of the GCL in TBI mice 5 weeks after injury. (**d**) The representative drawings of neuron dendritic trees in the GCL of sham mice, the inner layer of the GCL in TBI mice, and the outer layer of the GCL in TBI mice. (**e**) The quantification data shows that both the correctly migrated neurons and mis-located neurons in TBI mice had a significantly fewer number of branches, less total dendrite length, and shorter dendrite average length when compared to sham. Compared to the neurons in the inner layer of the GCL in TBI mice, the mis-located ones showed a dramatic reduction in the total dendrite length. (**f**) Sholl analysis also shows reduced dendritic complexity in both mis-located neurons and in normal position neurons in TBI mice compared to sham. (*p < 0.05, **p < 0.01, ***p < 0.001).

We further determined whether misplacement of neurons affects their morphological development by assessing their dendrite arborization. To trace the fate and morphological development of adult-born neurons in the hippocampus following TBI, we took advantage of red fluorescent protein (RFP)-expressing retrovirus, which can infect and integrate into the chromosomes of dividing cells and ectopically express RFP in the infected cells. This approach marks the birthdays of the cells and allows the investigator to trace the fate and morphological development of the individual neurons based on the RFP expression. The RFP retroviruses were transcranially injected into hippocampal hilus 1 day before receiving moderate TBI injury or sham-surgery. The retrovirus infected cells mainly are actively proliferating neural precursor cells (NPCs). The actively proliferating NPCs usually divide a few times before differentiating into immature neurons. Thus, an injection of retroviruses 1 day before TBI will ensure the labeled newborn immature neurons will go through the TBI-injured environment in the hippocampus. For those RFP labeled immature neurons that survived for 1 month after TBI, we assessed their positions in the GCL and their dendrite arborization using immunostaining with antibody against RFP (Fig. 5).

We found that the RFP expressing cells located at the inner GCL and exhibited typical mature neuronal morphology with extended dendrite arborizations that projected towards the molecular layer (ML) in the hippocampus of sham-surgery mice. Each RFP labeled mature neuron in the sham-surgery mice had an average of 9.33 ± 0.65 branches; the total length of a dendrite was 959.11 ± 106.07 μm; the average length of each dendrite branch was 103.41 ± 8.91 μm. In the TBI-injured animals, the RFP labeled mature neurons had reduced dendrite arborizations. It is worth noting that these neurons suffered in the injured hippocampus when they were newly born as immature neurons and survived for 1 month before animals were sacrificed. When the RFP labeled mature neurons located at the proper position at the inner one-third of the GCL in the hippocampus of TBI mice, in each neuron, the average of total dendrite branches was 6.83 ± 0.54; the total length of dendrites was 468.29 ± 66.65 μm; the average length of each dendrite branch was 70.17 ± 11.24 μm. The differences in the total number of dendrite branches, the total length of dendrites, and the average length of each dendrite branch were significantly smaller compared to control neurons. The Sholl analysis also showed a significant reduction in dendrite complexity which was evident between 60 μm to 140 μm from the cell body (Fig. 5f). When the RFP labeled mature neurons located at a misplaced position at the outer two-thirds of the GCL in the hippocampus of TBI mice, their impairment in morphological development was even greater. In each neuron, the average of total dendrite branches was further reduced to 3.8 ± 0.73; the total length of dendrites was 183.01 ± 39.9 μm; the average length of each dendrite was 48.79 ± 8.43 μm. The Sholl analysis also showed further reduction in dendrite complexity, which was evident between 40 μm to 140 μm compared to the sham control (Fig. 5f). These data indicated that TBI dramatically impairs the morphological development of immature neurons that survived the injured environment in the hippocampus, where TBI induced significant immature neuron death^39^.

## Discussion

TBI primarily causes cell death in the cortex. In addition, it induces secondary cell death in the hippocampus, leading to substantial cognitive impairment, motor dysfunction, and often epilepsy^2^. Cell replacement therapy is one of the active research areas for repairing damaged brain following TBI. Our previous reports showed that moderate TBI rapidly induced immature neuron death within hours after injury^39^,^40^. The immature neurons predominately die of necrosis within 24 hours but a low level of cell death in the hippocampal dentate gyrus can last as long as 2 weeks^40^. The highest density of dead cells in the hippocampus was found in the epicenter. From there it gradually decreased rostrally for at least 540 microns and caudally for at least 720 microns^40^. Here we further showed that, at the moderate level of TBI, a large number of those surviving immature neurons misplaced at the outer granular cell layer (GCL) of the hippocampal dentate gyrus (HDG), likely due to aberrant migration and failure to stop migrating at the appropriate position. Villasana *et al.* also reported that TBI increased dispersion of immature granular cells^41^. The aberrant migration of immature neurons occurs a few days after moderate TBI and lasts for 8 weeks (Fig. 2). At the peak of aberrant migration, about 50% of the immature neurons fail to stop migrating within the inner one-third of granular cell layer, continue migrating cross the borderline between the inner one-third of the GCL, and misplace at the outer two-thirds of the GCL (Fig. 2j). Misplaced immature neurons were found almost across the whole hippocampal dentate gyrus (Fig. 1e). We did not find obvious aberrant migration of immature neurons in the hippocampal dentate gyrus following mild TBI, but aberrant migration was dramatic following severe TBI (Data not shown). These immature neurons can specify into granular neurons at the ectopic position, but their morphological development was dramatically impaired with less dendritic complexity.

Neuronal migration is one of the major differences between regeneration in the CNS and in other organs. All newborn neurons in the CNS are required to migrate from their birthplace to their final destination to develop into functional neurons. Precisely regulated directional migration is required to ensure normal patterning, including position, lamination, orientation, neuronal subtype specification, target connection, and activity-dependent refinement^34^,^42^. Thus, migration is a ubiquitous feature that determines the final allocation of neurons in the nervous system, bringing cells into appropriate spatial relationships and establishing the basis for the subsequent wiring of neural circuitry. Aberrant migration may result in neurons misplaced at the wrong position, making inappropriate connections with nearby targets in the pre-existing neural networks, and performing inappropriate functions. Thus, aberrant migration has been shown to cause certain disorders such as epilepsy, dyslexia, and schizophrenia^30^.

The arrangement of neurons and axon projections in layers is a fundamental organizational principle in the mammalian hippocampus. The granular neurons in different laminae of the HDG received neural inputs from neurons of different regions in the brain. The dendrites of granular neurons locating at the inner one-third of the GCL mainly distribute at the molecular layer immediately adjacent to the granular cell layer. They form synapses with and receive input from ipsilateral and contralateral hippocampal commissural and associational fibers. While the dendrites of granular neurons locating at the outer two-thirds of GCL mainly distribute at the outer molecular layer, and form synapses with fibers from the entorhinal cortex^43^,^44^,^45^. Here we showed that there are large numbers of adult-born neurons misplaced at the outer GCL, instead of the inner GCL, in the hippocampus following TBI. Normally, a majority of adult-born neurons located at the inner GCL in the hippocampus will receive input from hippocampal commissural and associational fibers. However, when they misplaced in the outer GCL, they more likely received input from the entorhinal cortex instead. Improper integration of these adult born neurons following TBI may lead to functional impairment of learning and memory, which is one of the major complaints from patients who have suffered TBI^46^,^47^,^48^.

The molecular mechanisms on how TBI causes displacement of immature neurons in the outer GCL of adult hippocampus are completely unknown. Several molecules are found to govern neuronal migration in the cortex at the early embryonic development^30^, however the molecular mechanisms guiding neuronal migration in the postnatal brains are poorly understood. A limited number of genes have been shown to be involved in regulating neuronal migration in the subventricular zone (SVZ) of postnatal brain. Among them, we and other groups have reported that Slit, a secreted molecule, is the directional guidance molecule of newborn neurons migrating from SVZ to olfactory bulb^49^,^50^,^51^,^52^,^53^. But, the molecular mechanism guiding the migration and positioning of newborn neurons in the postnatal hippocampal dentate gyrus is poorly understood. Recently, it is shown that Disrupted-In-Schizophrenia 1 is required for relaying positional signals. However, it did not directly mediate neuronal migration^54^. Neuronal migration integrates multiple cellular and molecular events that enable immature neurons to move across the brain in an appropriate direction and to reach their final destination. It will be interesting to study whether the extracellular guidance cue is disregulated, and/or the immature neurons are less responsive to guidance cues causes immature neurons to fail to stop at the appropriate position. It will be also interesting to study whether neuroinflammation plays a role in alternating the immature neuron migration following TBI. By understanding these molecular mechanisms, it might be possible to find a way to prevent the mis-migration of immature neurons, and enhance functional regeneration following TBI.

TBI not only caused misplacement of adult-born neurons, it also impaired their dendrite development. Dendrites provide a massive membrane surface for the post-synaptic spines and act to conduct the electrochemical stimulation received from other neurons to the cell body. Reduction of dendrite arborization will significantly impair its critical roles in integrating the synaptic input and disrupt the signaling transduction for producing action potential by the neurons, which subsequently contributes to post-traumatic deficits in learning and memory.

Altogether, TBI affects multiple steps in the process of neurogenesis, including neural stem cell proliferation^12^,^13^,^14^,^15^,^16^,^17^,^18^,^19^,^25^,^55^,^56^,^57^,^58^, immature neuron death^39^, aberrant immature neuronal migration (Figs 1 and 2), mature neuron position (Fig. 3), dendrite development (Fig. 5), and functional integration into the existing neural network in the hippocampus^59^,^60^,^61^. Further understanding the molecular mechanisms of how TBI reshapes neurogenesis may provide approaches to promote functional repair of the damaged brain.

## Materials and Methods

### Animals

Male C57 BL/6 mice (Jackson Laboratories) were group-housed with a 12/12 light/dark cycle and free access to food and water ad libitum. They were used in experiments at an age of 12 weeks. All procedures were performed under protocols approved by the Animal Care and Use Committee at the Indiana University. All experiments were performed in accordance with guidelines and regulations of Indiana University Biosafety Committee.

### Controlled Cortical Impact Traumatic Brain Injury

C57 BL/6 mice at 12 weeks old were subjected to a controlled cortical impact (CCI) to produce a moderate level of injury to the brain as previously described^25^,^28^,^29^,^39^,^40^,^62^,^63^,^64^,^65^ using electromagnetic model. Briefly, the mice were anesthetized with 2.5% Avertin and placed in a stereotaxic frame (Kopf Instruments, Tujunga, CA) prior to TBI. Using sterile procedures, the skin was retracted and a 4 mm craniotomy centered between the lambda and bregma sutures was performed. A point was identified midway between the lambda and bregma sutures and midway between the central suture and the temporalis muscle laterally. The skullcap was carefully removed without disruption of the underlying dura. Prior to the injury, the head of the animal was angled on a medial to lateral plane so that the impacting tip was perpendicular to the exposed cortical surface. This was accomplished by rotating the entire stereotaxic frame on the transverse plane while leaving the nose bar at 5.0. The 3.0 mm impacting tip was used and the contact velocity was set at 3.0 m/sec with 1.0 mm amount of deformation. Sham (non-injured) animals received craniotomy, but no CCI injury.

### Tissue Processing

Animals were deeply anesthetized and then perfused transcardially with cold 0.9% saline, followed by a fixative containing 4% paraformaldehyde (PFA) in PBS. The brains were removed and post-fixed in PFA overnight, and then cryoprotected with 30% sucrose for 48 hours. Serial coronal sections (30 μm thick) were cut using a cryostat (Leica CM 1950) and stored at −20 °C. The sections were then processed for immunohistochemical analysis.

### Tracing the Fate of Proliferating Neural Stem/Progenitor Cells Following TBI

The C57/BL6 male mice at 12 weeks old were subjected to moderate TBI or sham surgery as indicated above. The sham mice and injured mice (5 for each) were given bromodeoxyuridine (BrdU) injections once a day for 7 days (50 mg/kg in 0.9% saline, i.p.; Sigma, St. Louis, MO). Four weeks after final injection the brains were fixed to evaluate the differentiation of BrdU-labeled cells in the hippocampus.

### Retrovirus Mediated Single New-Born Neuron Labeling

Retrovirus vector, CAG-RFP, was a kind gift from Dr. Gage in the Salk Institute for Biological Studies. The vector has red fluorescent protein (RFP) expression driven by CAG promoter. The retrovirus was produced by our viral core at the Department of Pharmacology and Toxicology, Indiana University. One day before TBI or sham surgery, retroviruses (2 μm of 1.7 × 10^7^ pfu/mL) were stereotaxically injected into the hilus. 4 weeks after TBI, the mice were sacrificed to assess the morphology of newborn neurons in the hippocampal dentate gyrus.

### Immunohistochemistry

To assess position of immature neurons in the hippocampus at different time points after TBI, 1 section from the epicenter of each animal were selected and processed to immunostaining with antibody against Dcx. Briefly, free-floating sections were washed with PBS 3 times and then incubated in blocking solution (0.1% Triton X-100, 1% bovine serum albumin and 5% normal goat serum in PBS) for 1 hour at room temperature, followed by an overnight incubation with primary antibody at 4 °C. Sections were then washed with PBS 3 times and incubated with the secondary antibody at room temperature for 2 hours. After being treated with DAPI for 2 minutes, the sections were washed with PBS 3 times and mounted on the slides. When the mounted sections dried, Fluoromount G was applied for preserving the fluorescence. To quantify distribution of misplaced immature neurons in the whole hippocampus, series of every sixth sections (180 μm apart, 12 sections in total) from each animal collected at 1 week after TBI were processed to immunostaining with antibody against Dcx. To compare mis-migration of immature neurons in the ventral and dorsal blades of the hippocampus, 1 epicenter section from each animal collected at 1 week after TBI was evaluated. To evaluate the position of post-injury born mature neurons, 3 epicenter sections from each animal were processed for immunostaining with antibodies against BrdU and NeuN. For the BrdU staining, pretreatment was performed. Free-floating sections were incubated in 2N HCl for 1 hour at room temperature, and then soaked in 0.1 M borate buffer for 10 min (pH 8.4). After being washed with PBS 3 times, the sections were processed according to standard blocking protocol as described above. Primary antibodies and their final concentrations were as follows: anti-BrdU antibody (1:400, rat, Accurate Chemical and Scientific), anti-NeuN antibody (1:1000, mouse, Millipore), anti-Prox1 antibody (1:1000, rabbit, Millipore), anti-Dcx antibody (1:1000, Guinea pig, Millipore), and anti-RFP antibody (1:1000, chicken, Abcam). Secondary antibodies from Jackson ImmunoResearch Laboratories, Inc., were all applied with same dilution of 1:1000.

### Microscopy

The sections were analyzed by light microscopy with an inverted microscopy system (Zeiss Axiovert 200 M) interfaced with a digital camera (Zeiss Axio Cam MRc5) controlled by a computer. Images were captured in software (AxioVision, v4.0) and assembled and labeled in Photoshop 7.0 (Adobe Systems). Images with Dcx-positive cells, BrdU-positive cells, or BrdU and cell type specific marker double-labeled cells in the DG were captured through all the sections.

### Image analysis

#### Quantifying the position of immature neurons in the hippocampus

The granular cell layer was divided into the inner one-third and outer two-thirds (white line shown in Fig. 1a1 and cartoon in Fig. 1c). The numbers of Dcx-positive immature neurons in both regions were counted. Data was expressed as inner percentage (The numbers of Dcx-positive immature located in the inner one-third of the granular cell layer/the total number Dcx-positive immature counted) or outer percentage (The numbers of Dcx-positive immature located in the outer two-thirds of the granular cell layer/the total number Dcx-positive immature counted).

#### Quantifying the migratory distance of immature neurons in the hippocampus

The migratory distance of each immature neuron was determined by measuring the distances between the center of their cell bodies and the borderline of the subgranular zone and the hilus (Fig. 1d).

#### Dendrite Quantification

For each RFP-positive neuron, all branches of the dendritic tree were reconstructed at 20x magnification using a motorized microscope (Zeiss Imager M2) with Neurolucida software (Microbrightfield, VT). A 3D analysis of the reconstructed neurons was performed using NeuroExplorer software (Microbrightfield). More than 10 neurons were studied for each of the 4 animals in TBI or sham control group. The branch data of neurons from the same animal were averaged. Several aspects of dendritic morphology were examined. The complexity of dendrite trees was assessed with the Sholl analysis^66^,^67^. The number of intersections of dendrites was calculated with concentric spheres positioned at radial intervals of 20 μm.

### Statistical analysis

For statistical analysis, data were analyzed using a suitable analysis of variance, followed by Fisher’s LSD as *post hoc* test if required. Data was expressed as mean ± standard error. SPSS software was used and significance was set at p < 0.05. Details of the analysis used for each group of data were listed in the supplemental Table 1.

## Additional Information

**How to cite this article**: Ibrahim, S. *et al.* Traumatic Brain Injury Causes Aberrant Migration of Adult-Born Neurons in the Hippocampus. *Sci. Rep.* **6**, 21793; doi: 10.1038/srep21793 (2016).

## Supplementary Material

Supplemental table 1
