# Supplementary material for: Traumatic Brain Injury Causes Aberrant Migration of Adult-Born Neurons in the Hippocampus
Source: Sci Rep. 2016 Feb 22;6:21793. doi: 10.1038/srep21793 (PMC4761898; doi:10.1038/srep21793)
Supplement: Supplemental table 1 [file srep21793-s1.pdf]

Traumatic Brain Injury Causes Aberrant Migration of Adult-Born Neurons in the Hippocampus

Sara Ibrahim[1] , Weipeng Hu[2], Xiaoting Wang[1], Xiang Gao[1], Chunyan He[3,4], Jinhui Chen[1,4,5]

Supplemental Table-1 Statistical analysis

	Type of analysis	Subjects	Source	F value	p value
Fig. 1 c1	two-way ANOVA	Percentage of Dcx-positive cells	Position	$F_{(1,18)}$=482.288	<0.001
			Injury	$F_{(1,18)}$=0	1
			Interaction	$F_{(1,18)}$=627.548	<0.001
Fig. 1 d1	two-way ANOVA	Percentage of Dcx-positive cells	Distance	$F_{(2,17)}$=110.73	<0.001
			Injury	$F_{(1,18)}$=0	1
			Interaction	$F_{(2,17)}$=137.066	<0.001
Fig. 1 e	one-way ANOVA	Percentage of Dcx-positive cells in the outer GCL	injury	$F_{(12,29)}$=35.905	<0.001
Fig. 1 f	student's t test	Percentage of Dcx-positive cells in the outer GCL	injury	Not applicable	0.465
Fig. 2 j	one-way ANOVA	Percentage of Dcx-positive cells in the outer GCL	injury	$F_{(8,35)}$=65.364	<0.001
Fig. 2 k	two-way ANOVA	Percentage of Dcx-positive cells	Distance	$F_{(2,129)}$=455.410	<0.001
			Injury time	$F_{(8,123)}$=0	1
			Interaction	$F_{(16,115)}$=42.543	<0.001
Fig. 3 e	two-way ANOVA	Percentage of BrdU and NeuN double-positive cells	Position	$F_{(1,18)}$=79.507	<0.001
			Injury	$F_{(1,18)}$=0	1
			Interaction	$F_{(1,18)}$=102.757	<0.001
Fig. 5 e	one-way ANOVA	Total dendrite length	injury	$F_{(2,17)}$=18.546	<0.001
		Dendrite branch	injury	$F_{(2,17)}$=17.051	<0.001
		Average dendrite length	injury	$F_{(2,17)}$=8.075	0.003
Fig. 5 f	repeated measures ANOVA	Intercross	Injury	$F_{(2,24)}$=15.530	0.001
			Radium	F=18.681	<0.001
			Interaction	F=5.984	<0.001
